# TLQP-21 facilitates diabetic wound healing by inducing angiogenesis through alleviating high glucose-induced injuries on endothelial progenitor cells

**DOI:** 10.1007/s00210-023-02808-8

**Published:** 2024-01-06

**Authors:** Yaqi Fang, Yuexia Zhu, Minxia Zhang, Hua Ying, Yubo Xing

**Affiliations:** 1 Laboratory Medicine Center, Department of Clinical Laboratory, Affiliated People’s Hospital, Zhejiang Provincial People’s Hospital, Hangzhou Medical College, No. 158, Shangtang Road, Gongshu District, Hangzhou Zhejiang, 310053 China; 2 Department of Endocrinology, Affiliated People’s Hospital, Key Laboratory of Endocrine Gland Diseases of Zhejiang Province, Zhejiang Provincial People’s Hospital, Hangzhou Medical College, No. 158, Shangtang Road, Gongshu District, Hangzhou Zhejiang, 310053 China

**Keywords:** Diabetic wound, TLQP-21, Endothelial progenitor cells, PI3K

## Abstract

Diabetes mellitus (DM) is a metabolic disease with multiple complications, including diabetic cutaneous wounds, which lacks effective treating strategies and severely influences the patients’ life. Endothelial progenitor cells (EPCs) are reported to participate in maintaining the normal function of blood vessels, which plays a critical role in diabetic wound healing. TLQP-21 is a VGF-derived peptide with promising therapeutic functions on DM. Herein, the protective effects of TLQP-21 on diabetic cutaneous wound and the underlying mechanism will be investigated. Cutaneous wound model was established in T2DM mice, followed by administering 120 nmol/kg and 240 nmol/kg TLQP-21 once a day for 12 days. Decreased wound closure, reduced number of capillaries and EPCs, declined tube formation function of EPCs, and inactivated PI3K/AKT/eNOS signaling in EPCs were observed in T2DM mice, which were sharply alleviated by TLQP-21. Normal EPCs were extracted from mice and stimulated by high glucose (HG), followed by incubated with TLQP-21 in the presence or absence of LY294002, an inhibitor of PI3K. The declined cell viability, increased apoptotic rate, reduced number of migrated cells, declined migration distance, repressed tube formation function, and inactivated PI3K/AKT/eNOS signaling observed in HG-treated EPCs were markedly reversed by TLQP-21, which were dramatically abolished by the co-culture of LY294002. Collectively, TLQP-21 facilitated diabetic wound healing by inducing angiogenesis through alleviating HG-induced injuries on EPCs.

**Supplementary Information:**

The online version contains supplementary material available at 10.1007/s00210-023-02808-8.

## Introduction

DM is a metabolic disease characterized by inappropriate hyperglycemia, which is a major public health burden worldwide. The global prevalence of DM is projected to reach 4.4% by 2030, making it the fastest growing global health epidemic (NCD-RisC 2016). Endothelial dysfunction caused by diabetes mellitus is the key and initiating factor of diabetic vascular complications. Endothelial dysfunction in diabetic macrovascular complications is characterized by reduced nitric oxide (NO) release, enhanced oxidative stress, increased production of inflammatory factors, abnormal angiogenesis, and impaired endothelial repair (Knapp et al. 2019). The effects of DM on the endothelium involve multiple alterations, including hyperglycemia, fatty acid oxidation, reduced NO, oxidative stress, inflammatory activation, and altered barrier function. Therefore, the restoration of vascular endothelial dysfunction is the key for the treatment of diabetic vascular complications.

The innermost layer of blood vessels is constituted by vascular endothelial cells (EC), which regulate vascular homeostasis and angiogenesis by producing and secreting a variety of vasoactive substances and growth factors (Michiels 2003). Bone marrow-derived endothelial progenitor cells (EPCs) have been recognized as endogenous cells that contribute to endothelial repair and angiogenesis (Asahara et al. 1999). The EPC functions are impaired to varying degrees in diabetic patients, such as cell adhesion, cell migration, and cell incorporation into vascular structures. The adhesion of EPCs to collagen, fibronectin and human umbilical vein endothelial cells (HUVECs) is found to be hindered in patients with type 2 diabetes (Tepper et al. 2002). Ischemic lesions and endothelial dysfunction are common in diabetes, which can be repaired by EPCs (Pysna et al. 2019). Thus, Enhancing the function of EPCs will be a promising method for treating diabetic vascular complications.

VGF is a neuropeptide precursor genetically encoding 68 kDa, consisting of 615 (human) or 617 (rat, mouse) residues, which is ubiquitously expressed in the central nervous system (CNS), peripheral nervous system (PNS), and various endocrine systems (Levi et al. 2004). TLQP-21 is one of the VGF-derived peptides, which is composed of 21 amino acids. TLQP represents the four N-terminal amino acid sequences of the peptide, namely Thr-Leu-Gln-Pro (Bresciani et al. 2019). It is reported that TLQP-21 plays a critical role in diabetes, and shares multiple common properties with glucagon-like peptide-1 (GLP-1) receptor agonists that have been widely used in the treatment of diabetes, such as enhancing glucose-stimulated insulin secretion, improving blood glucose control, and reducing islet cell apoptosis (Doyle and Egan 2007). In addition, TLQP-21 is reported to reduce side effects of GLP-1, such as nausea, vomiting, abdominal distension, and loss of appetite, which will lead to treatment discontinuation in severe situation (Sun et al. 2012). TLQP-21 may be a targeted drug to enhance islet β cell survival and function, and alleviates the development of type 2 DM by enhancing islet β cell survival and function (Stephens et al. 2012). However, whether TLQP-21 has such a direct effect on vascular endothelial cells in diabetic patients remains unknown. The present study aimed to investigate the protective effects of TLQP-21 on diabetic vascular endothelial dysfunction and the underlying mechanisms.

## Materials and methods

### The establishment of cutaneous wounds in diabetic mice and grouping

Male db/db mice (7–9 weeks, n = 18) and male db/m mice (7–9 weeks, n = 6) were purchased from SHANGHAI SLAC LABORATORY ANIMAL CO. LTD (China). For the establishment of cutaneous wounds in db/db mice, anesthesia was induced by intraperitoneal injection of 40 mg/kg sodium pentobarbital, and all hair was removed from the back with an electric razor and depilatory cream. A 1 cm × l cm full-thickness skin defect wound was made on the median caudal side of the back of mice, and attention was paid to thoroughly remove the subcutaneous carnosus layer until the superficial surface of the sarcolemma to prevent the wound from shrinking. Animal were divided into Control, T2DM, 120 nmol/kg TLQP-21 and 240 nmol/kg TLQP-21 groups. After cutaneous wound modeling, db/db mice TLQP-21 treatment groups were intraperitoneally injected with 120 nmol/kg and 240 nmol/kg TLQP-21 once a day for 12 days, respectively, while db/db mice in the T2DM group were intraperitoneally injected with the same volume of normal saline once a day for 12 days. In the control group, db/m mice were intraperitoneally injected with the same volume of normal saline once a day for 12 days.

### The measurement of wound closure

On the 0, 4, 8, and 12 days post the first treatment, images of wounds were taken using an inverted microscope (AE2000, Motic, China) and the image J software was used to analyze the wound area and calculate the percentage of wound healing. The formula was as follows: wound closure (%) = (initial wound area- wound area on the observation day)/initial wound area × 100%.

### Immunohistochemical assay

Wounds were collected from mice at 12 days post the first treatment. Platelet endothelial cell adhesion molecule (CD31) staining was used to assess the state of angiogenesis. Skin samples from the injured area and surrounding tissue, about 1 cm in diameter and 2 mm in thickness, were cut and fixed in 10% formalin for 6 h at room temperature. Samples were subsequently embedded in paraffin. After dewaxing, sections were rehydrated with a decreasing alcohol series, followed by incubating in 10 mM citrate buffer at 90° C for 30 min. After blocking with 5% serum for 3 h at room temperature, slides were incubated with anti-CD31 antibody (1:100, AF6191, Affinity, USA) for 1 h at room temperature, followed by 1 h incubation with biotinylated secondary antibody (1:3000, ab97080, Abcam, USA). Samples were counterstained with hematoxylin for 2 min at room temperature, followed by taking images using the inverted microscope (AE2000, Motic, China). CD31-positive tubular structures were considered to be capillaries and the number of capillaries was counted.

### The detection on the number of EPCs in the peripheral blood using the flow cytometry

Peripheral blood was collected in lithium heparin tubes after adequate anesthesia with 1% sodium pentobarbital. 100 mL of anticoagulant blood was incubated with FITC-conjugated anti-mouse Sca‑1 (1:200, 11-5981-82, ThermoFisher, USA) and PE-conjugated anti-mouse Flk‑1 antibodies (1:200, 12-5821-82, ThermoFisher, USA) for 30 min at 4° C in the dark, and then blood cells were lysed using red cell lysis buffer. After washing twice with PBS, cells were resuspended in 400 µL of PBS, and the Sca‑1+/Flk‑1 + cell ratio was analyzed by flow cytometry (NovoCyte, Agilent, USA).

### The isolation and incubation of EPCs

Mice were sacrificed under anesthesia, followed by sterilizing the skin of the abdomen and separating the bilateral tibia and femur of the hind limbs. The surrounding muscles, tendons and connective tissue were removed, and the epiphysis at both ends was cut off under sterile conditions, with the bone marrow cavity fully exposed. The bone marrow cavity was washed repeatedly by PBS in a 5 mL syringe until it turned white. The bone marrow suspension was collected in a 15 mL centrifuge tube, centrifuged at a low speed of 1000 r/min for 5 min, and the supernatant was discarded. EGM-2-MV medium was added to resuspend cells, and the cell density was adjusted to 1 × 10^6^ cells /mL for inoculation. After 2 days, the non-adherent cells were discarded, and the adherent cells were cultured in a new EGM-2-MV medium. The medium was changed once every 3 days, and the growth of cells was observed under an inverted microscope (AE2000, Motic, China). Extracted EPCs were identified by determining the percentage of Sca‑1+/Flk‑1 + cells using the flow cytometry (NovoCyte, Agilent, USA).

### Tube formation assay

A total of 40,000 EPCs were seeded in 96-well plates precoated with 50 µL/ well of growth factor-induced matrigel. After 8 h of incubation at 37° C, morphological images of the tubes were captured using a computer-assisted microscope (AE2000, Motic, China). The number of tubes and the average tube length were determined based on images.

### Western blotting assay

EPCs were collected to extract total proteins, followed by quantified using the BCA method. After conducting the separation with the 12% SDS-PAGE, proteins were transferred to the PVDF membrane, which was then blocked in the 5% skim milk. The primary antibodies against p-PI3K (1:1000, AF3241, Affinity, USA), PI3K (1:1000, AF6241, Affinity, USA), p-AKT (1:1000, AF0016, Affinity, USA), AKT (1:1000, AF6259, Affinity, USA), eNOS (1:1000, AF6247, Affinity, USA), p-eNOS (1:1000, AF3247, Affinity, USA), and GAPDH (1:10000, 10494-1-AP, Proteintech, USA) were added, followed by incubation with the secondary antibody (1:6000, 7074, CST, USA). After incubation for 1 h, the ECL solution was added for exposure and the expression level was quantified with the Image J software.

### Cell culture and treatments

Normal EPCs were extracted from the mouse as described above, which were cultured in EGM2-MV medium containing high concentration of glucose (HG, 40 mM) for 24 h to establish the in vitro model. EPCs cultured in EGM2-MV medium containing 5.5 mM glucose were taken as the negative control. HG treated EPCs were incubated with 50 nM TLQP-21 (Zhang et al. 2013) in the presence or absence of 10 µM LY294002 (Duan et al. 2019).

### CCK-8 assay

EPCs were implanted in wells and 10 µL of CCK8 reagent was introduced after adhesion, followed by incubation for 2 h. The absorbance value of each well was measured at a wavelength of 450 nm by a microplate reader (CMaxPlus, MD, USA).

### Apoptosis detection using the flow cytometry

EPCs were implanted in the 6-well plate and cultured for 48 h, which were resuspended and introduced with 10 µL Annexin V reagent and 5 µL PI reagent in the apoptosis kit (556,547, BD, USA). After 10 min incubation, EPCs were centrifugated and resuspended using PBS buffer in flow assay tubes, which were loaded onto the flow cytometry (NovoCyte, Agilent, USA) for apoptosis detection.

### Transwell assay

In the lower chamber, 0.5 mL complete medium was added, while the upper chamber was loaded with cell suspension, which were cultured at 37° C for 12 h. After discarding the upper chamber, the chamber was rinsed with PBS and transferred to the well of a 24-well plate pre-added with 700 µL 4% formaldehyde. After fixing for 20 min, 700 µL of 0.1% crystal violet was introduced. Images were taken under an inverted microscope (AE2000, Motic, China).

### Wound healing assay

EPCs were seeded in 6-well plates to reach 90% density, which were scraped with a yellow pipet. The scratch area was washed twice with PBS to remove floating cells. The scratch width was recorded at 0 h. 24 h later, the wound was photographed again and the scratch width at 24 h was recorded, followed by calculating the migration distance.

### Immunofluorescence assay

EPCs were fixed using the 4% paraformaldehyde. 0.5%Triton X-100 was permeated at room temperature for 20 min, followed by adding 5% BSA at 37° C for 30 min. The primary antibody against CD31 (1:200, ab222783, Abcam, UK) was then added and incubated at 37° C for 3 h. Fluorescence secondary antibody (1:500, ab150080, Abcam, UK) was introduced and incubated at 37° C for half an hour in the dark. DAPI was then dropped, followed by incubation at 37° C in the dark for 5 min to dye the nucleus. The petri dish was sealed with 50% glycerol, and then the images were observed and collected under a fluorescence microscope (Ts2-FC, Nikon, Tokyo, Japan).

### Statistical analysis

Data were presented using mean ± SD and analyzed with the one-way ANOVA method using the GraphPad prism software 6.0. *P* < 0.05 was considered a significant difference.

## Results

### TLQP-21 alleviated the cutaneous wounds in diabetic mice

**Fig. 1:**
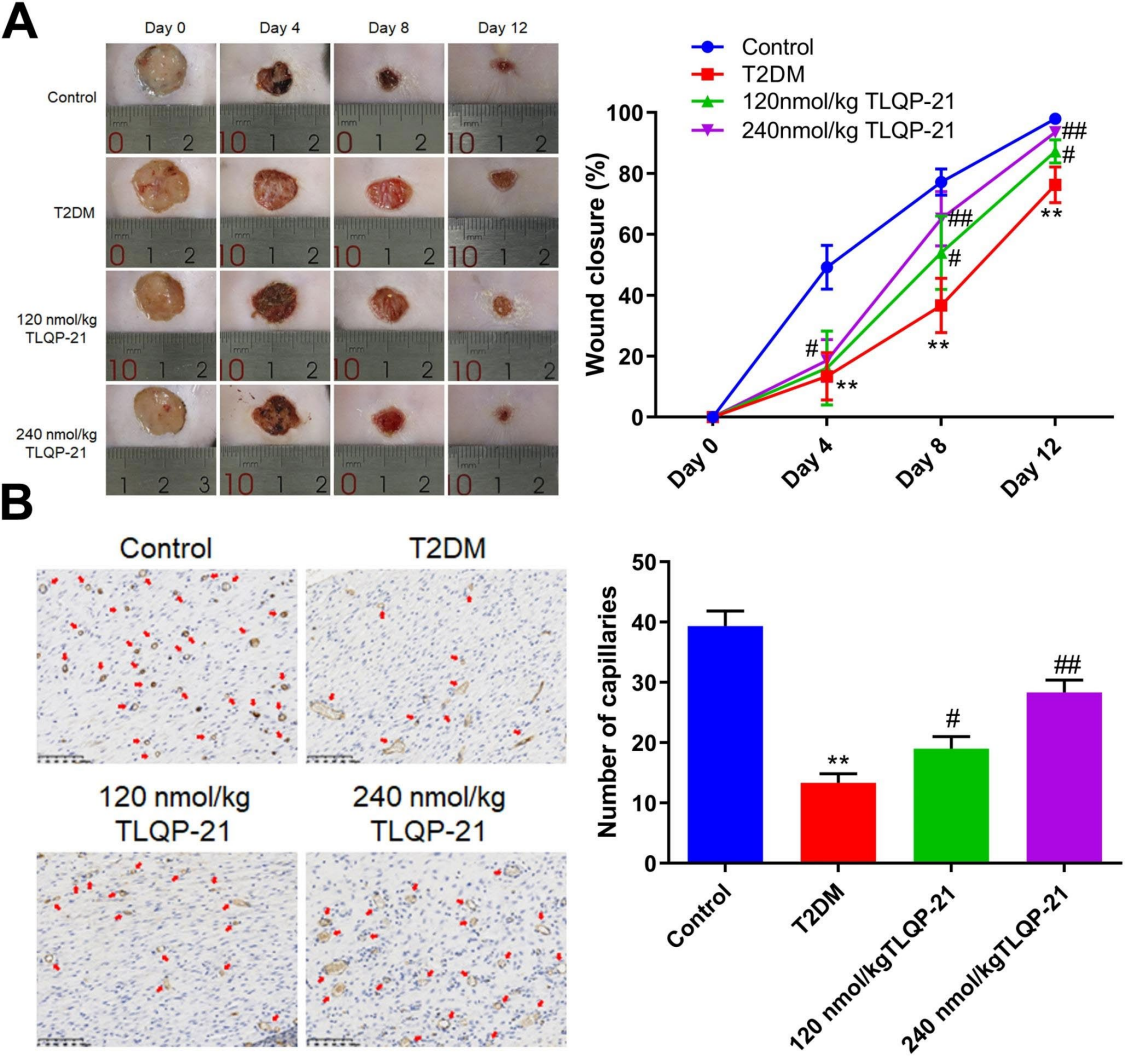
The cutaneous wounds in diabetic mice were healed by TLQP-21. **A** Images of wounds and wound closure on the 0, 4, 8, and 12 days posting administration. **B** The expression of CD31 in wound tissues was detected by the immunohistochemical assay and the number of capillaries was counted (**p < 0.01 vs. control, #*p* < 0.05 vs. T2DM, ##*p* < 0.01 vs. T2DM)

Cutaneous wounds were introduced on T2DM mice, followed by administered with 120 and 240 nmol/kg TLQP-21 for 12 days, respectively. Representative images of wounds in each group were shown in Fig. 1A. On the 8th day, the wound closure was markedly reduced from 77.2 to 36.7% in the T2DM group, which was sharply increased to 54.0% and 65.1% by 120 and 240 nmol/kg TLQP-21, respectively. Moreover, on the 12th day, the wound closure in the control, T2DM, 120 nmol/kg TLQP-21, and 240 nmol/kg TLQP-21 groups was 98.0%, 76.3%, 92.2%, and 93.5%, respectively. Furthermore, the number of capillaries in the T2DM group was signally decreased from 39.3 to 13.3, which was greatly increased to 19.0 and 28.3 by 120 and 240 nmol/kg TLQP-21, respectively (Fig. 1B). On the 12th day, the data of body weight, food intake, and fasting blood glucose levels in each group were shown in Fig. S1. The body weight, food intake, and fasting blood glucose levels in the T2DM group were markedly increased, which were minorly changed by 120 nmol/kg TLQP-21 and dramatically repressed by 240 nmol/kg TLQP-21.

### TLQP-21 increased the percentage of EPCs in diabetic mice

**Fig. 2:**
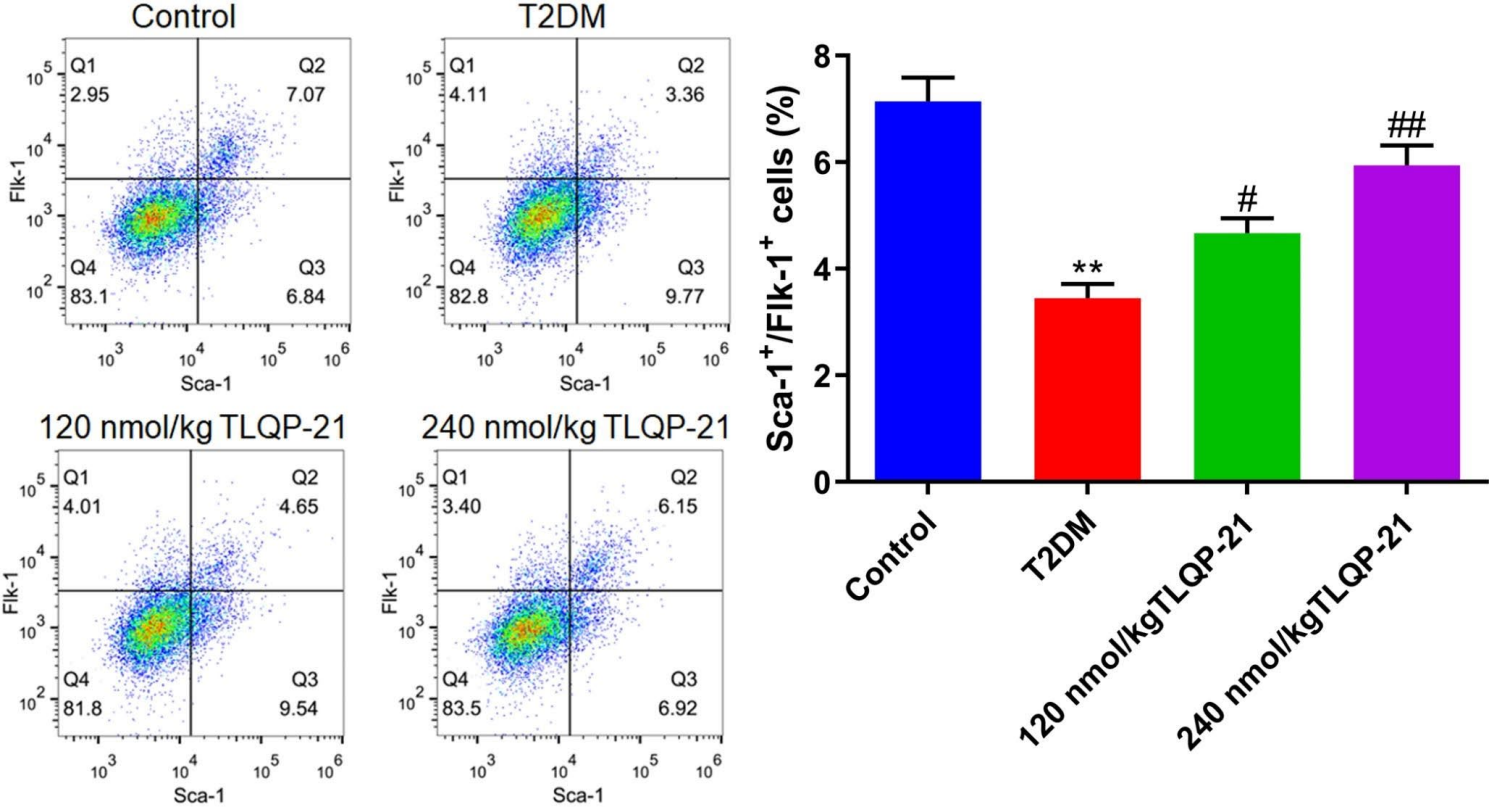
The number of peripheral EPCs in diabetic mice was increased by TLQP-21. The percentage of Sca‑1+/Flk‑1 + cells in the peripheral blood was evaluated by the flow cytometry (**p < 0.01 vs. control, #*p* < 0.05 vs. T2DM, ##*p* < 0.01 vs. T2DM)

To explore the influence of TLQP-21 on the number of EPCs in diabetic mice, the proportion of EPCs in the peripheral blood was determined. The percentage of Sca‑1+/Flk‑1 + cells in the T2DM group was declined from 7.14 to 3.45%, which was markedly elevated to 4.67% and 5.94% by 120 and 240 nmol/kg TLQP-21, respectively (Fig. 2).

### TLQP-21 enhanced the tube formation function of EPCs in diabetic mice

**Fig. 3:**
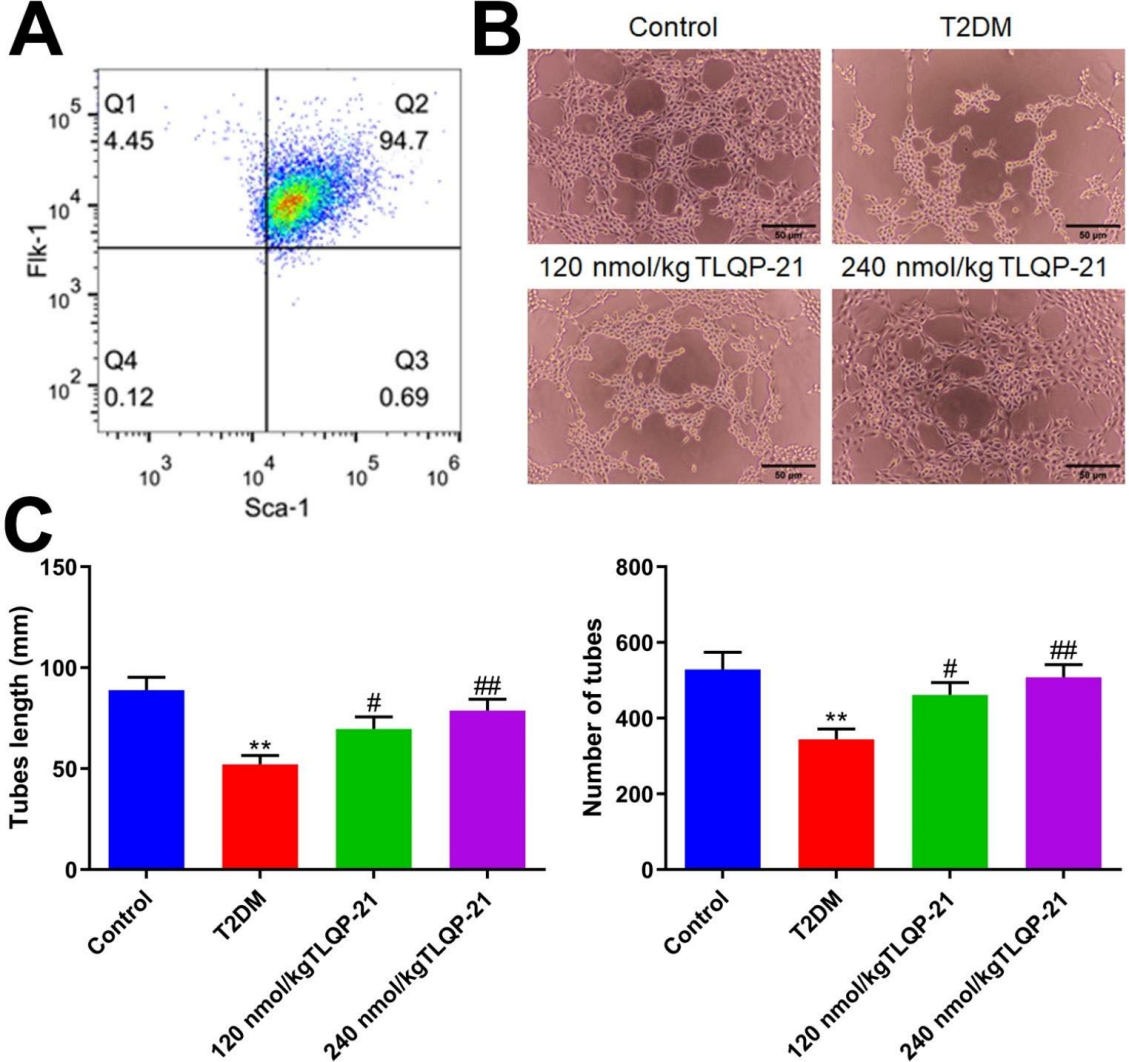
TLQP-21 enhanced the tube formation function of EPCs in diabetic mice. **A** The purity of isolated EPCs was determined by the flow cytometry. **B** Representative images of tubes formed by EPCs. **C** The tube lengths were determined and the number of tubes were counted (**p < 0.01 vs. control, #*p* < 0.05 vs. T2DM, ##*p* < 0.01 vs. T2DM)

To explore the impact of TLQP-21 on the function of EPCs in diabetic mice, EPCs were extracted from each mouse. Firstly, the percentage of Sca‑1+/Flk‑1 + ratio in extracted cells was 94.7%, indicating that a high purity of EPCs were extracted from mice (Fig. 3A). Representative images of tubes formed by EPCs in each group was shown in Fig. 3B. The average tube length in the control, T2DM, 120 nmol/kg TLQP-21, and 240 nmol/kg TLQP-21 groups was 88.9, 52.1, 69.7, and 78.8 mm. Moreover, the number of tubes in the T2DM group was decreased from 529.0 to 344.0, which was sharply increased to 461.3 and 508.3 by 120 and 240 nmol/kg TLQP-21, respectively (Fig. 3C).

### TLQP-21 activated the PI3K/AKT/eNOS signaling in EPCs of diabetic mice

**Fig. 4:**
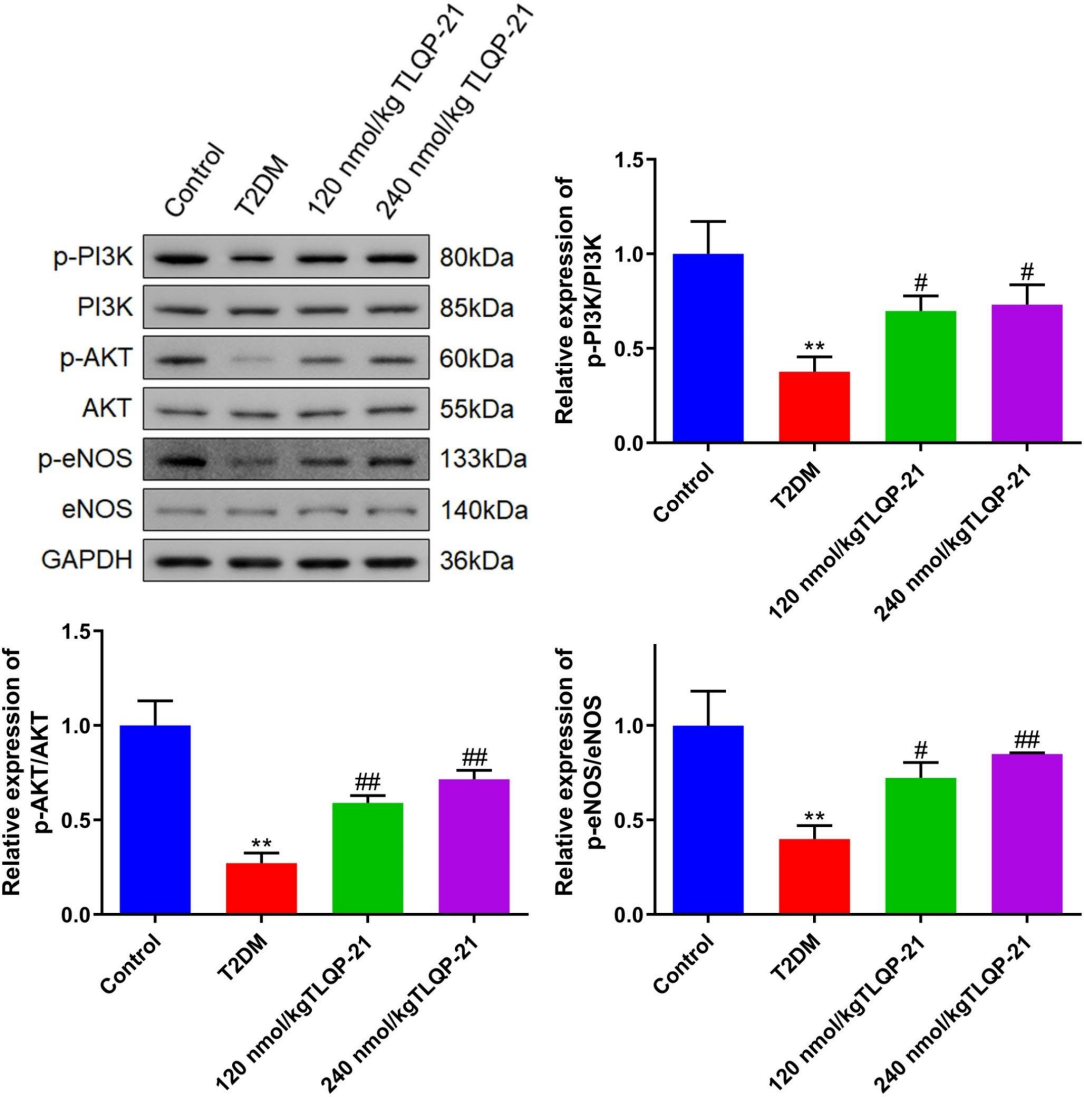
The PI3K/AKT/eNOS signaling in EPCs of diabetic mice was activated by TLQP-21. The expression level of p-PI3K, PI3K, p-AKT, AKT, p-eNOS, and eNOS in EPCs was detected by the Western blotting assay (**p < 0.01 vs. control, #*p* < 0.05 vs. T2DM, ##*p* < 0.01 vs. T2DM)

To explore the potential regulatory mechanism of TLQP-21 on the proliferation and function of EPCs, the activity of PI3K/AKT/eNOS signaling was evaluated. Levels of p-PI3K/PI3K, p-AKT/AKT, and p-eNOS/eNOS were markedly reduced in the T2DM group, which were signally elevated by 120 and 240 nmol/kg TLQP-21 (Fig. 4), implying an activating effect of TLQP-21 on the PI3K/AKT/eNOS signaling in EPCs.

### TLQP-21 ameliorated HG-induced injury in EPCs by activating the PI3K signaling

**Fig. 5:**
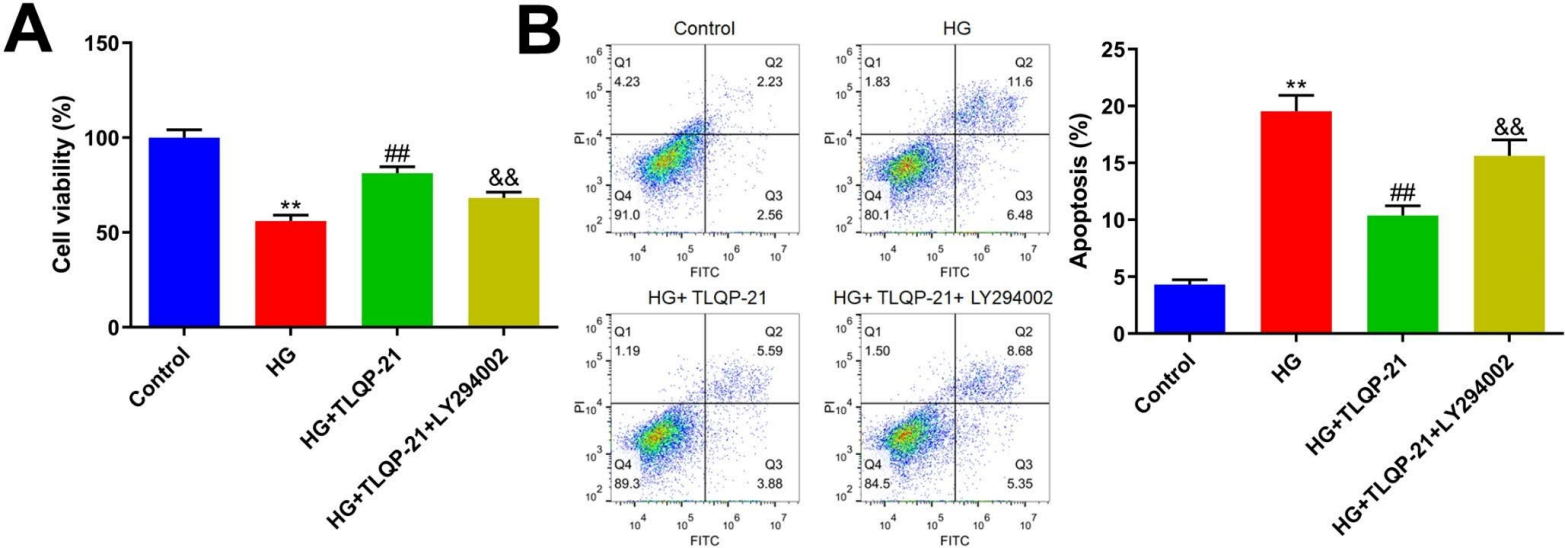
TLQP-21 ameliorated HG-induced injury in EPCs by activating the PI3K signaling. **A** The cell viability was determined by the CCK-8 assay. **B** The apoptosis of ECPs was detected by the flow cytometry (**p < 0.01 vs. control, ##*p* < 0.01 vs. HG, &&*p* < 0.01 vs. HG + TLQP-21)

EPCs were treated with HG, followed by incubated with TLQP-21 in the presence or absence of LY294002, an inhibitor of PI3K. The cell viability of EPCs was found signally reduced from 100.0 to 56.1% by HG, which was sharply increased to 81.3% by LY294002. After the co-culture of LY294002, the cell viability of EPCs was greatly reduced to 68.3% (Fig. 5A). Furthermore, the apoptotic rate in the control, HG, HG + TLQP-21, and HG + TLQP-21 + LY294002 groups was 4.3%, 19.6%, 10.4%, and 15.6%, respectively (Fig. 5B).

### TLQP-21 repaired the reduced migration function of HG-treated EPCs by activating the PI3K signaling

**Fig. 6:**
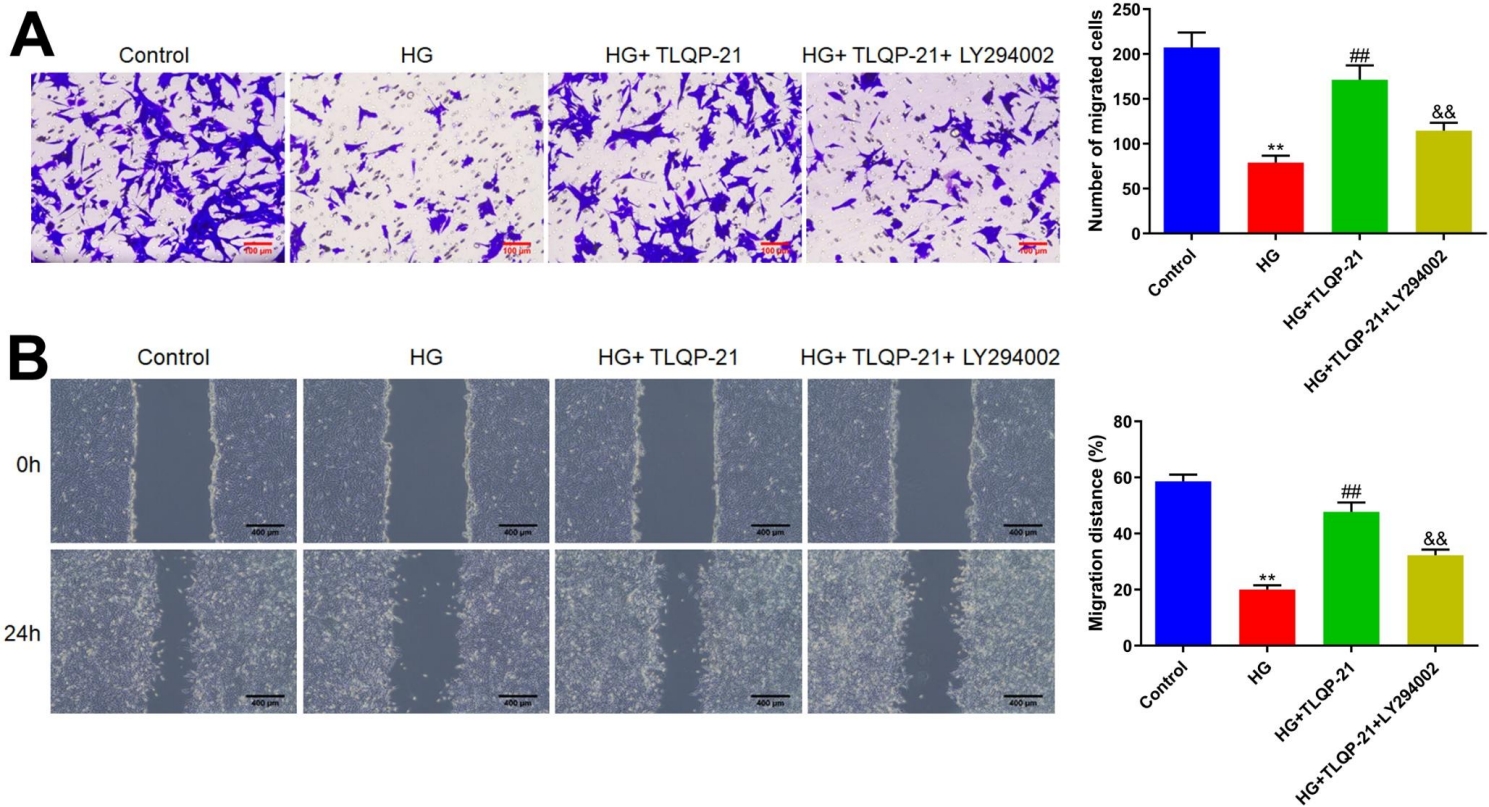
The reduced migration function of HG-treated EPCs was repaired by TLQP-21 via activating the PI3K signaling. **A** The migration ability of EPCs was detected by the Transwell assay. **B** The migration distance of EPCs was determined by the wound healing assay (**p < 0.01 vs. control, ##*p* < 0.01 vs. HG, &&*p* < 0.01 vs. HG + TLQP-21)

The migration function of EPCs was further investigated. In the Transwell assay, the number of migration cells in the control, HG, HG + TLQP-21, and HG + TLQP-21 + LY294002 groups was 207.3, 79.0, 171.3, and 114.7, respectively (Fig. 6A). Furthermore, in the wound healing assay, the migration distance of EPCs was declined from 58.7 to 20.1%, which was sharply increased to 47.7% by TLQP-21. After the co-culture of LY294002, the migration distance of EPCs was markedly reduced to 32.3% (Fig. 6B).

### TLQP-21 alleviated the damaged tube formation function of HG-treated EPCs by activating the PI3K signaling

**Fig. 7:**
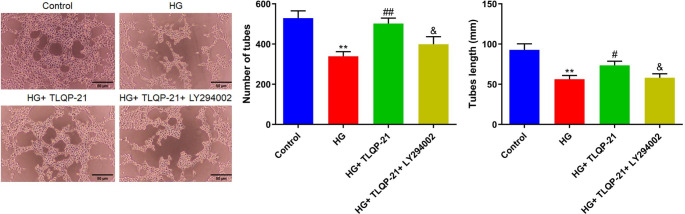
TLQP-21 alleviated the damaged tube formation function of HG-treated EPCs by activating the PI3K signaling. The tube formation ability of EPCs was evaluated, the tube lengths were determined, and the number of tubes were counted (**p < 0.01 vs. control, #*p* < 0.05 vs. HG, ##*p* < 0.01 vs. HG, &*p* < 0.05 vs. HG + TLQP-21)

Subsequently, the tube formation assay was conducted. The number of tubes in the HG group was decreased from 529.3 to 339.3, which was greatly elevated to 502.0 by TLQP-21. After the co-culture of LY294002, the number of tubes was reduced to 399.0. Furthermore, the average tube length in the control, HG, HG + TLQP-21, and HG + TLQP-21 + LY294002 groups was 92.8, 56.3, 73.6, and 58.2 mm, respectively (Fig. 7).

**Fig. 8:**
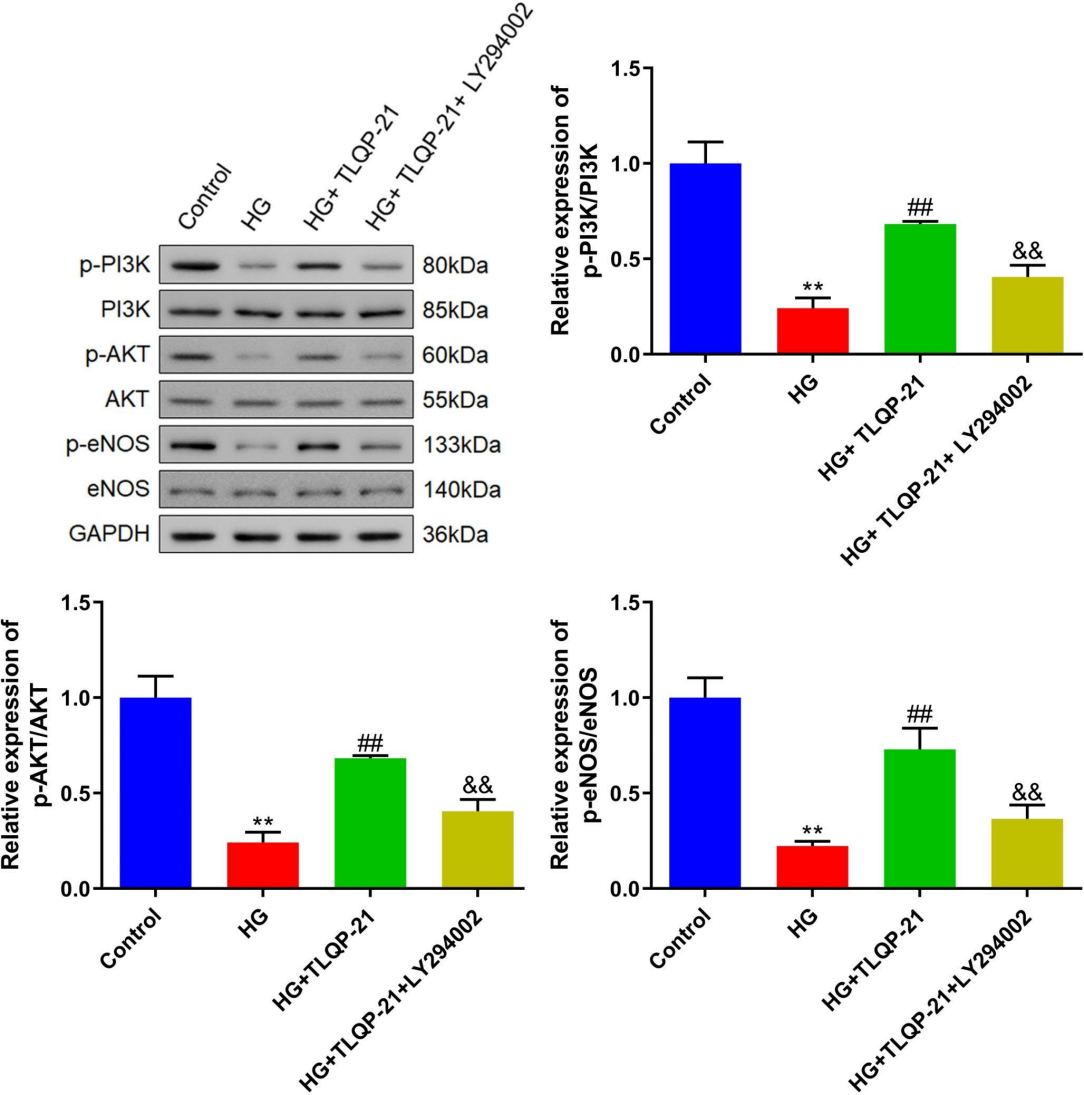
TLQP-21 alleviated the PI3K/AKT/eNOS signaling in HG-treated EPCs. The expression level of p-PI3K, PI3K, p-AKT, AKT, p-eNOS, and eNOS in EPCs was determined by the Western blotting assay (**p < 0.01 vs. control, ##*p* < 0.01 vs. HG, &&*p* < 0.01 vs. HG + TLQP-21)

### TLQP-21 alleviated the PI3K/AKT/eNOS signaling in HG-treated EPCs

In HG-treated EPCs, levels of p-PI3K/PI3K, p-AKT/AKT, and p-eNOS/eNOS were markedly reduced, which were sharply increased by TLQP-21. Following the co-culture of LY294002, levels of p-PI3K/PI3K, p-AKT/AKT, and p-eNOS/eNOS were dramatically repressed (Fig 8).

### TLQP-21 did not show impacts on the differentiation of EPCs to endothelial cells (ECs)

To check the impact of TLQP-21 on the differentiation of EPCs, the level of CD31 in each group was checked. As shown in Fig S2, CD31 was markedly downregulated in the HG group. However, compared to the HG group, the CD31 level was slightly altered by TLQP-21. Compared to the HG + TLQP-21 group, the CD31 level was markedly repressed in the HG + TLQP-21 + LY294002, suggesting that TLQP-21 did not show impacts on the differentiation of EPCs to ECs. However, HG or LY294002 dramatically suppressed the differentiation of EPCs to ECs.

## Discussion

The skin of diabetic patients is easily damaged, with delayed healing process, which finally contributes to the development of stubborn and refractory ulcer and diabetic foot. With characteristics of high infection rate, slow healing process, and high disability rate, diabetic wound lacks effective clinical treatments (Shao et al. 2020; Zubair and Ahmad 2019). Therefore, the treatment of diabetic wounds is one of the important issues that need to be solved in clinic. Normal wound healing is a complex and orderly biological process, which includes three stages of inflammatory response (cell proliferation, tissue maturation and reconstruction), involving a variety of cells, mediators, growth factors, and extracellular matrix (Wan et al. 2022a, b; Zhang et al. 2015). Specificities of diabetic wound, including high glucose microenvironment of wound tissue, peripheral neurovascular disease, excessive inflammation, and reduced migration, proliferation, and tube formation ability of vascular endothelial cells, contribute to the difficulty of diabetic ulcer healing. Among them, diabetic wound neovascularization disorder is the most important factor for poor wound healing (Zubair and Ahmad 2019; Fukui et al. 2017). Herein, the cutaneous wound model was established in mice, identified by decreased wound closure and reduced number of capillaries, which were in line with the research reported by Guo et al. (2020). After the administration of TLQP-21, the wound closure and number of capillaries were markedly reversed, implying a repayment function of TLQP-21 on wound in diabetic mice.

Healthy vascular ECs maintain the integrity of blood vessels and are the basis for blood vessels to complete their functions. Normal vascular endothelium possesses the ability of self-repairment. However, long-term high glucose and high lipid environment will repeatedly damage vascular ECs, which also contributes to the declined number of EPCs in peripheral blood (Pysna et al. 2019). These repeated injuries and stimuli will lead to chronic pathological changes in blood vessels. Poor blood supply in the affected area will be resulted from the hardened wall and narrow lumen, forming a vicious circle and eventually leading to pathological changes (Menegazzo et al. 2012). The expansion of neovascularization in wound granulation tissue mainly depends on the activation, proliferation, and migration, of EPCs from healthy tissues around the wound, and the participation of EPCs from bone marrow (Kaushik and Das 2019). EPCs are precursor cells that can differentiate into vascular endothelial cells and participate in blood vessel formation, which are important cornerstones for the construction and maintenance of endothelial vessels and the function of most human systems (Wan et al. 2022a, b). By studying the animal model of skin transplantation, it has been confirmed that the mechanism of angiogenesis in the transplanted tissue is mainly related to the re-proliferation of EPCs in the host (Park et al. 2017). Transplantation of EPCs from human peripheral blood into mouse skin wounds is found to promote wound healing and increase the infiltration of monocytes/macrophages, suggesting that EPCs promote wound healing by recruitment of a large number of inflammatory cells and increasing neovascularization (Suh et al. 2005). In the nude mouse model, the infusion of mouse EPCs is beneficial to the angiogenesis and healing of the wound (Lee et al. 2018). Herein, the number of EPCs in the peripheral blood of diabetic mice and the tube formation function of EPCs were marked repressed, which were consistent with results presented by Han et al. (2017). After the administration of TLQP-21, the number of peripheral EPCs and the tube formation function of EPCs were markedly repaired, implying that the protection of TLQP-21 on diabetic wound might be correlated with the repaired EPCs function. Furthermore, the proliferation, migration, and tube formation function of EPCs were sharply repressed by HG stimulation, which were similar to data reported by Li et al. (2020). After the incubation of TLQP-21, the impaired proliferation, migration, and tube formation function of EPCs were signally alleviated, which further confirmed the influence of TLQP-21 on the EPCs function.

PI3K/AKT/eNOS signaling pathway plays an important role in EPCs mobilization and function improvement (Everaert et al. 2010; Chen et al. 2013; Yu et al. 2010; Zheng et al. 2007). Activated PI3K induces the production of the second messenger PIP3 at the plasma membrane, which binds to AKT and PDK1 that contain intracellular PH domain. The activated AKT subsequently regulates the cell proliferation, differentiation, apoptosis, and migration by activating or inhibiting a series of downstream substrates such as caspase9, NF-κB, and eNOS (Wang et al. 2022). Several drugs, such as hepatocyte growth factor and pinocembrin, have been shown to inhibit EPCs apoptosis or improve the number and function of EPCs by activating the PI3K/AKT/eNOS signaling pathway (Yu et al. 2010; Zheng et al. 2007; Yang et al. 2013). Herein, the PI3K/AKT/eNOS axis was found markedly inhibited in EPCs of diabetic mice, which were markedly activated by TLQP-21, suggesting that the function of TLQP-21 on EPCs might be correlated to the activity of the PI3K/AKT/eNOS signaling. Furthermore, the regulatory effect of TLQP-21 on the HG-induced impaired proliferation, migration, and tube formation function of EPCs was abolished by LY294002, an inhibitor of the PI3K/AKT/eNOS signaling, which further confirmed that TLQP-21 enhanced the function of EPCs by activating the PI3K/AKT/eNOS signaling. In the future work, the regulatory mechanism will be further confirmed by co-administering diabetic wound mice with both TLQP-21 and LY294002. Furthermore, there are limitations in the present study. Firstly, as revealed in Fig S1, there was a possibility that the treatment of TLQP-21 improved wound healing via the amelioration of diabetic conditions, at least in a part, in diabetic mice. The protective property of TLQP-21 might be a combination of antidiabetic and anti-angiogenesis function. In addition, the previous report (Zhang et al. 2013) raised the possibilities that TLQP-21 has the protective effects on the differentiated endothelial cells against HG exposures. Considering influence of TLQP-21 on the CD31 expression in HG-treated EPCs shown in Fig S2, results with HG-treated EPCs presented in the present study might include outcomes from differentiated endothelial cells, at least in a part.

Collectively, TLQP-21 facilitated diabetic wound healing by inducing angiogenesis through alleviating HG-induced injuries on EPCs.

### Electronic supplementary material

Below is the link to the electronic supplementary material.

Supplementary Material 1

## Data Availability

The datasets used and/or analyzed during the current study are available from the corresponding author on reasonable request.
