# Supplementary material for: TLQP-21 facilitates diabetic wound healing by inducing angiogenesis through alleviating high glucose-induced injuries on endothelial progenitor cells
Source: Naunyn Schmiedebergs Arch Pharmacol. 2024 Jan 6;397(7):4993–5004. doi: 10.1007/s00210-023-02808-8 (PMC11166834; doi:10.1007/s00210-023-02808-8)
Supplement: Supplementary file 1 — Supplementary Material 1 [file 210_2023_2808_MOESM1_ESM.docx]

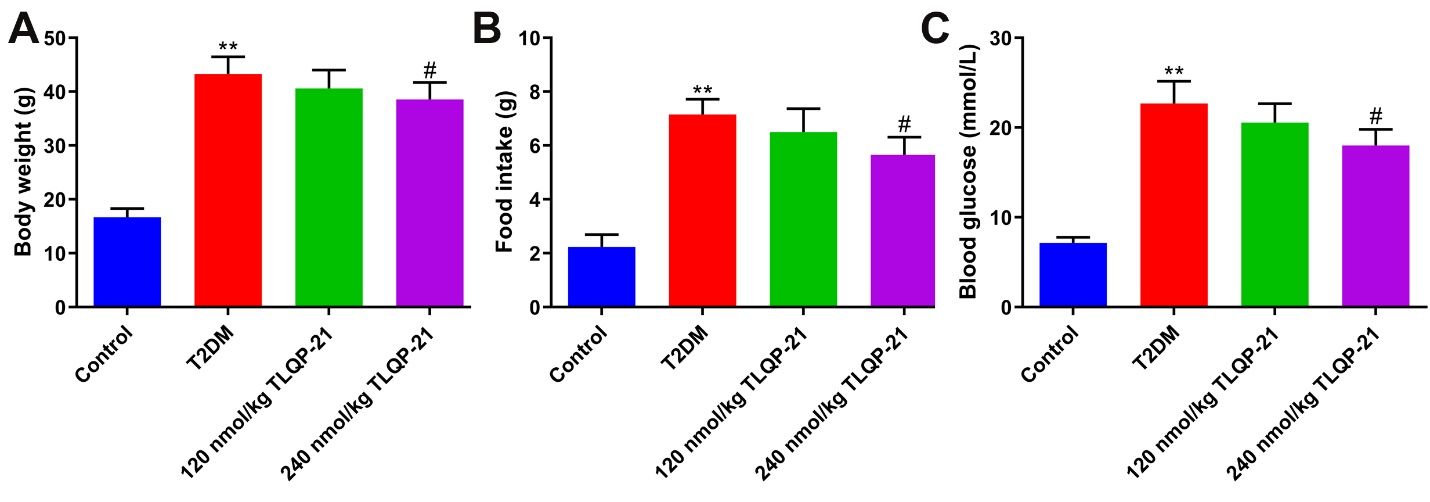


Fig S1. TLQP-21 slightly repressed the body weight (A), food intake (B), and fasting blood glucose levels (C) in T2DM mice (**p<0.01 vs. control, #p<0.05 vs. T2DM).


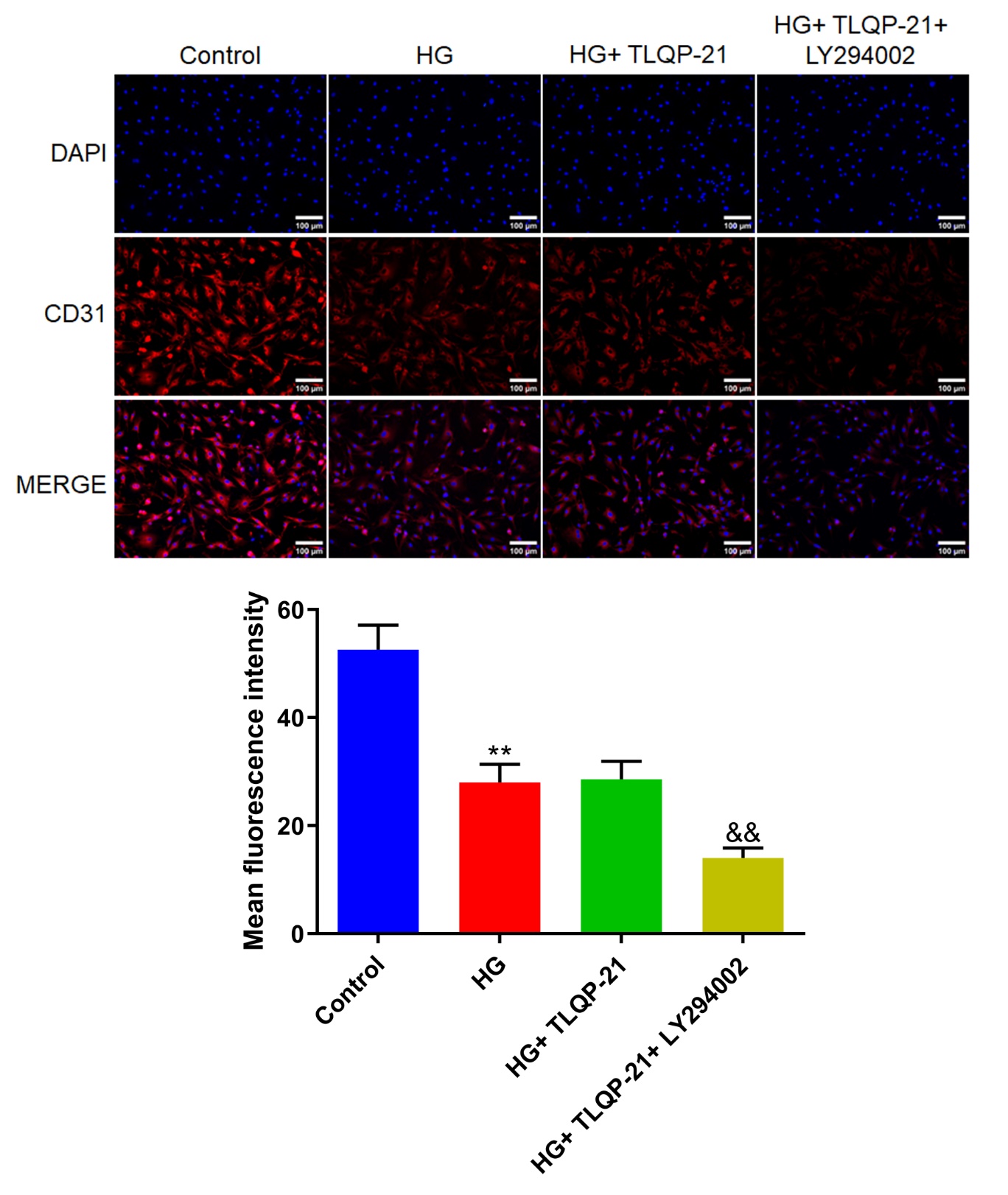


Fig S2. TLQP-21 did not show impacts on the differentiation of EPCs to ECs. The expression of CD31 in each group was detected using the immunofluorescence assay (**p<0.01 vs. control, &&p<0.01 vs. HG+ TLQP-21).
